# Microarray-Based RNA Profiling of Breast Cancer: Batch Effect Removal Improves Cross-Platform Consistency

**DOI:** 10.1155/2014/651751

**Published:** 2014-07-03

**Authors:** Martin J. Larsen, Mads Thomassen, Qihua Tan, Kristina P. Sørensen, Torben A. Kruse

**Affiliations:** ^1^Department of Clinical Genetics, Odense University Hospital, Sdr. Boulevard 29, 5000 Odense C, Denmark; ^2^Human Genetics, Institute of Clinical Research, University of Southern Denmark, Winsløwvej 19, 5000 Odense C, Denmark; ^3^Epidemiology, Biostatistics and Biodemography, Institute of Public Health, University of Southern Denmark, J.B. Winsløws Vej 9B, 5000 Odense C, Denmark

## Abstract

Microarray is a powerful technique used extensively for gene expression analysis. Different technologies are available, but lack of standardization makes it challenging to compare and integrate data. Furthermore, batch-related biases within datasets are common but often not tackled. We have analyzed the same 234 breast cancers on two different microarray platforms. One dataset contained known batch-effects associated with the fabrication procedure used. The aim was to assess the significance of correcting for systematic batch-effects when integrating data from different platforms. We here demonstrate the importance of detecting batch-effects and how tools, such as ComBat, can be used to successfully overcome such systematic variations in order to unmask essential biological signals. Batch adjustment was found to be particularly valuable in the detection of more delicate differences in gene expression. Furthermore, our results show that prober adjustment is essential for integration of gene expression data obtained from multiple sources. We show that high-variance genes are highly reproducibly expressed across platforms making them particularly well suited as biomarkers and for building gene signatures, exemplified by prediction of estrogen-receptor status and molecular subtypes. In conclusion, the study emphasizes the importance of utilizing proper batch adjustment methods when integrating data across different batches and platforms.

## 1. Introduction

The microarray technology has been extensively used for genome-wide gene expression analysis. Microarray is a well-established, cost-effective, high-throughput technology able to simultaneously measure the expression levels of thousands of genes and hereby offers an efficient way to generate a snapshot of the entire transcriptome. Several different microarray platforms are commercially available, differing with respect to the fabrication methodologies and length of the oligonucleotide probes. Some manufactures use photolithography (light directed) methods while others rely on ink-jet technology for* in situ* synthesis of oligonucleotides onto a solid array surface. The probes lengths typically vary from 25-mer to 60-mer. In addition to these commercially available platforms, spotted arrays with either cDNA or synthesized oligonucleotide probes deposited onto the array surface comprise a cost effective alternative. Spotted arrays can be either commercially manufactured or* in-house* manufactured. Because of their relatively low cost and flexibility, the spotted microarray technology has been widely used in many academic laboratories.

The workflow of a microarray gene expression analyses consists of multiple steps. First RNA is extracted, amplified, and either biotin-labeled or labeled with fluorescent dyes (Cy3/Cy5) depending on the platform. Subsequently, the labeled RNA is hybridized to the array, typically overnight. After hybridization, the arrays are washed and scanned. Finally, the scanned images are quantified by specialized software. All abovementioned steps comprise a potential source of systematic variation. In addition, there are multiple technical issues that must be controlled in the fabrication and use of spotted arrays. Spotted arrays are often manufactured in relatively small batches under semistandardized conditions. This often introduces systematic differences between the measurements of different batches, often termed “batch effects” [1]. Furthermore, lack of standardization makes it challenging to compare and integrate data from the different microarray technologies available as it introduced platform-dependent systematic variation. Several studies have demonstrated that pooling data derived from different platforms is a complex task with numerous pitfalls [2, 3]. Additional factors are known to introduce systematic variation including when the analysis is conducted at different sites, by alternating personnel, in different experiments, or simple due to day-to-day variation.

Combining data from different experiments/platform/batches without reducing possible systematic variant can give rise to misleading results that can be strong enough to mask or even confound true biological signals and lead to misinterpretation of the data. For unmasking, it is necessary to identify and remove the batch effects before proceeding. Several approaches have been developed for removal of systematic batch effects. Single value decomposition and principal component analysis (PCA) have been used by subtracting the component representing the batch effect from the data [4]. Distance-weighted discrimination (DWD) uses a modified version of the support vector machine (SVM) algorithm to correct for batch effect [5]. ComBat, proposed by Johnson et al., applies an empirical Bayes approach by pooling information across genes and shrinks the batch effect parameter toward the overall mean of the batch estimates across genes [6]. Other commonly used batch effect methods include mean centering, standardization, and ratio-based methods [7].

Due to its extensive use, thousands of gene expression microarray datasets have been deposited to public databases making these repositories valuable data sources. Microarray gene expression data has been widely used in medical decision-making research. For clinical use of array-based diagnostics, significant numbers of patient samples are needed for training and evaluation. Therefore, reuse, pooling, and integration of multiple microarray dataset are attractive approaches. Because of the heterogeneous nature of microarray datasets, it is important to address the issue of cross-platform variation as demonstrated in several studies when combining different dataset from different platforms analyzed in different laboratories, for example, medical decision-making purposes [7–9]. Furthermore, as the RNA sequencing technology develops and matures, another level of batch effects needs to be taken into consideration when datasets of different origin are to be pooled.

In the current study, we analyzed the same 234 breast cancers on two different microarray platforms, our* in-house* 29K array platform and Agilent SurePrint G3 microarray platform. The 29K dataset contained known batch effects associated with the fabrication procedure. Using these unique datasets, the aim was to assess the significance of removing systematic batch effects existing within a dataset and when integrating datasets of different platforms.

## 2. Materials and Methods

### 2.1. Ethics Statement

The study was carried out as a retrospective register study and in accordance with the Helsinki Declaration. The study has been approved by The National Committee on Health Research Ethics of Denmark (S-VF-20020142), waiving the requirement for informed consent for the study.

### 2.2. Patient Material

This study was performed on a series of 243 frozen primary breast tumors obtained from the biobanks of the Department of Pathology, Odense University Hospital and the Danish Breast Cancer Cooperative Group (DBCG). The tumor samples comprise a subset of a larger series of 253 tumor samples previously reported [10, 11]. Breast tumor tissues from 120 patients with germline mutations in* BRCA1* (*n* = 33) or* BRCA2* (*n* = 22) or familial non-*BRCA1/2* cases with no detectable germline mutation in* BRCA1* or* BRCA2* (*n* = 65) were included in the study. In addition, 123 primary tumor samples from sporadic breast cancers were included in the study. In order to determine the tumor cell content, slides of the frozen tumor biopsies were haematoxylin-eosin-stained and examined by a pathologist at the Department of Pathology, Odense University Hospital. Samples analyzed in the present study contained at least 50% tumor cells. Immunohistochemistry determined estrogen receptor (ER) expression data was obtained from the Danish Breast Cancer Cooperative Group (DBCG). Gene expression analyses were performed using two different microarray platforms: the* in-house* manufactured 29K oligonucleotide microarray platform and Agilent SurePrint G3 platform.

### 2.3. Microarray Fabrication

For manufacturing of the in-house spotted arrays, a human oligonucleotide library consisting of 28,919 DNA oligonucleotide probes (60-mer) was purchased from Compugen-Sigma-Genosys (The Woodlands). The oligonucleotide probes were solubilized in 150 mM sodium phosphate buffer (pH 8.5) to a final concentration of 20 pmol/*μ*L and spotted onto CodeLink HD (SurModics) activated glass slides by a high-precision spotting robot (Virtek ChipWriter Pro, ESI). The slides have an active polymer coating of amine-reactive groups permitting the 5′-amino modified DNA oligos to covalently attach under high relative humidity. The microarray fabrication was carried out in a controlled environment at 38% humidity and the temperature was held constant at 23°C. SMP 2.5 stealth pins (TeleChem International) was used to deposit the oligos onto surface of the slides. Up to 85 arrays were spotted per batch. After printing, slides were incubated overnight at 70% humidity and blocked as recommended by the manufacturer.

### 2.4. Gene Expression Analysis

Total RNA was extracted from freshly frozen tumor tissue. RNA extraction was carried out using Trizol Reagent (Invitrogen) followed by RNeasy Micro Kit (Qiagen) including DNase treatment. RNA concentration was determined using a NanoDrop Spectrophotometer (NanoDrop Technologies) and the quality assessed by the Agilent 2100 Bioanalyzer using Agilent RNA 6000 Nano Kit (Agilent Technologies). RNA integrity numbers (RIN) were calculated, and RNA was amplified and labeled using the Amino Allyl MessageAmp II aRNA Amplification Kit (Ambion) according to the manufacturer's protocol, starting with 750 ng of total RNA. Amplified aRNA from the tumor samples were labeled with Cy5. Universal Human Reference RNA (Stratagene) was labeled with Cy3 and used as reference RNA. Dye incorporation efficiency was measured by NanoDrop Spectrophotometer. Labeled sample aRNA corresponding to 200 pmol Cy5 and reference aRNA 130 pmol Cy3 were hybridized to the in-house spotted arrays, whereas 20 pmol Cy5 sample aRNA and 20 pmol Cy3 reference aRNA were used for hybridization to Agilent SurePrint G3 Human GE 8 × 60K Microarrays (Agilent Technologies). Fragmentation, hybridization, and washing for both spotted arrays and Agilent arrays were carried out using the Agilent Gene Expression Hybridization Kit and Gene Expression Wash Buffer Kit (Agilent Technologies) according to the manufacturer's protocol in a low ozone environment. Subsequently, the arrays were scanned using an Agilent G2565CA Microarray scanner (Agilent Technologies). Microarray data have been deposited into the Gene Expression Omnibus (GSE54275).

### 2.5. Data Preprocessing

The scanned images of the spotted arrays were quantified by Gene Pix Pro 6.0 (Molecular Devices), whereas images from Agilent arrays were processed by Agilent Feature Extraction Software 10.7.3.1 (Agilent Technologies). In the subsequent data preprocessing, raw intensity data from the spotted arrays and Agilent arrays were treated equally. Raw intensity data were background corrected using normexp method and bad quality features flagged during feature extraction were removed from further analysis [12]. Data were then within-array normalized by loess normalization method, and quantile normalization method was used for between-array normalization [13, 14]. The normalized values were used to calculate log_2_ transformed Cy5/Cy3 ratios. Data preprocessing was performed using the R package* limma* [15, 16]. Replicate probes were collapsed by calculating the median. Missing expression values were imputed by* k*-nearest neighbors averaging (*k* = 10). The Agilent dataset contained 0.012% missing data points, and the 29K dataset contained 1.144% missing data points.

### 2.6. Reannotation of Probe Sets

To obtain the overlapping gene set measured on both platforms and to perform an unbiased cross-platform comparison, reannotation of the probes was performed. For both the platforms, the probes were reannotated to gene symbols by Agilent eArray tool (http://earray.chem.agilent.com/earray) using the original probe sequences provided by the manufacturer. Based on the overlapping gene set, a data set was created for each platform. In cases of multiple probes per gene symbol, only the probe with the maximum mean intensity (based on Cy5 intensity values) was kept.

### 2.7. Systematic Bias Adjustments

The ComBat method was used for adjustment of the batch effects observed across the different spotting batches and to adjust for systematic and technical differences between the two platforms [6].

### 2.8. Unsupervised Methods

Principal component analysis (PCA) plots were generated using data where the expression level of each gene had been standardized to zero mean and unit variance by Qlucore Omics Explorer 2.3 (Qlucore).

### 2.9. Correlation and Agreement Analysis

To assess the correlation between sample pairs and between gene pairs from the two platforms, we calculated Pearson's correlation coefficients. To evaluate cross-platform reproducibility after pooling the two datasets, the intraclass correlation coefficients, ICC(1,1), were calculated. Wilcoxon rank sum test was used when comparing the correlation coefficients.

### 2.10. Detection of Differential Expressed Genes

Differential expressed gene analysis was carried out using significance analysis of microarrays (SAM) method using the implementation found in the R package* samr.*

### 2.11. Prediction of Estrogen Receptor Status

ER status was predicted by the gene expression data using the probes targeting the* ESR1* transcript. For each platform, the optimal sensitivity-specificity cutoff was determined based on receiver operating characteristic (ROC) curves, performed by the R package* pROC *[17]. The statistical significance was assessed by Fisher's exact statistic.

### 2.12. Molecular Subtype Classification

The tumors were classified into five intrinsic molecular subtypes using the 50-gene subtype classifier (PAM50) described by Parker et al. [18]. Distances to each of the subtype centroids defined by the PAM50 classifier were calculated using Spearman's rank correlation; hereby, the subtype classification was assigned based on the nearest of the centroids. All 50 genes comprising PAM50 could be mapped to the Agilent platform, whereas 49/50 genes were found on the spotted platform. Subtype prediction was performed using the R package* genefu *[19].

Cramer's *V* coefficient was used as a measure of the relative strength of the associations, ranging from 0 to 1 (perfect association), using the implementation found in the R package* vcd* [20].

## 3. Results and Discussion

### 3.1. Experimental Setup

**Figure 1:**
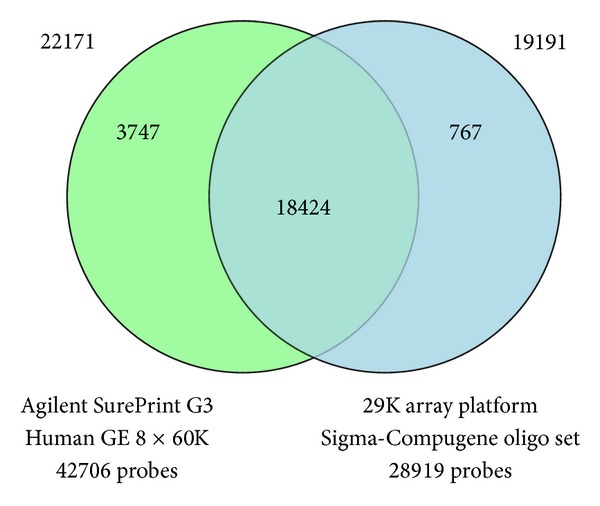
Venn-diagram illustrating the distribution of shared gene targets among the Agilent SurePrint G3 platform and the 29K array platform. 18424 gene symbols were found to be represented on both platforms.

The aim of the present study was to assess the significance of removing systematic batch effects existing within a dataset when integrating datasets of different platforms and perform a systematic cross-platform comparison of the 29K gene expression microarray platform and Agilent SurePrint G3 Human GE 8 × 60K Microarrays [21]. A series of 243 primary breast tumors (33* BRCA1*, 22* BRCA2*, 65 non-*BRCA1/2*, and 123 sporadic) were analyzed on both the 29K microarrays and Agilent microarrays. The gene expression analyses were conducted as two color analyses with a common reference sample cohybridized to all arrays. We sought to control other possible sources of variations that could influence the comparison and introduce additional nonplatform dependent differences. Thus, identical RNA amplification/labeling method and hybridization/washing conditions were used for both analyses. To avoid that day-to-day or batch-to-batch variation influenced the biological interpretation of the results of the RNA extraction, the RNA amplification steps and array hybridizations steps were performed in randomized sample orders. To provide the most up-to-date annotations, we reannotated all probe sequences and mapped them to gene symbols. To enable comparison of the two platforms, we created data subsets with only the shared gene set. In total, 22171 and 19191 unique gene symbols were represented on the Agilent arrays and spotted arrays, respectively, of which 18424 gene symbols are available on both arrays (Figure 1). Mapping to Entrez Gene IDs identified only 16673 overlapping Entrez Gene IDs. Thus, gene symbols were used for generation of the data subsets.

### 3.2. Uncovering and Eliminating Within-Experiment Batch Effects

**Figure 2:**
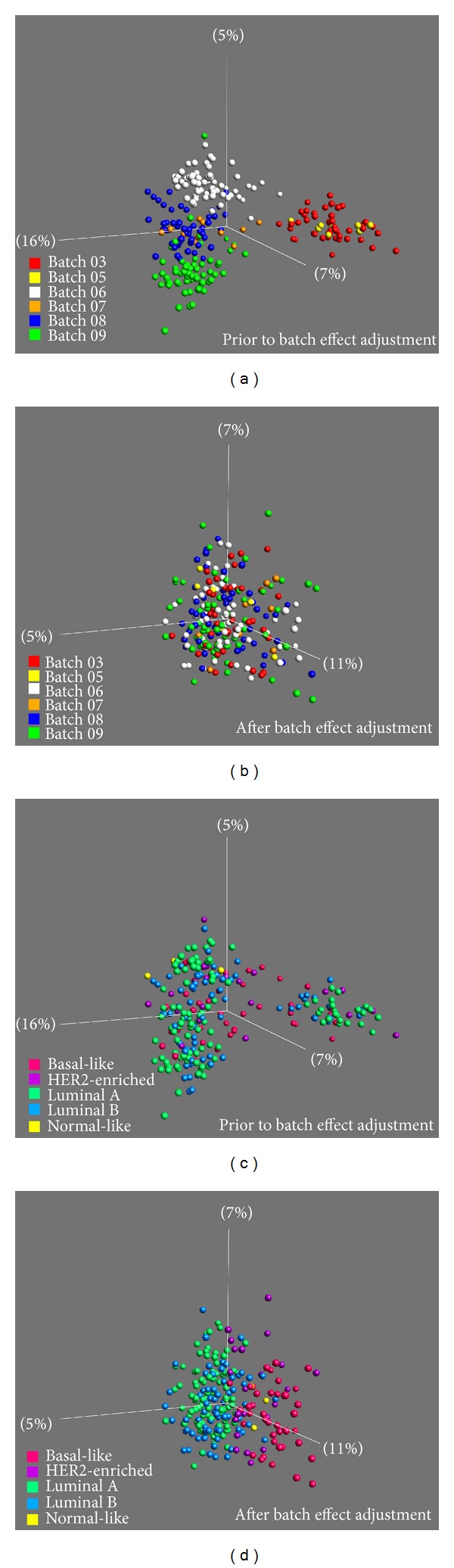
PCA plots showing all 243 samples analyzed using the 29K array platform prior to ((a) and (c)) and after batch-effect adjustment using ComBat method ((b) and (d)). Colors in (a) and (b) correspond to the different fabrication batches. Colors in (c) and (d) correspond to the molecular breast cancer subtypes classified using PAM50 subtype classifier.

The 29K arrays utilized in the present study were manufactured in 6 different batches (Table 1). Visualization of the 29K dataset by principal component analysis (PCA) revealed a high level of interbatch variations in the gene expression profiles (Figure 2(a)). To adjust for the batch-to-batch variations observed in the spotted dataset, the ComBat method proposed by Johnson et al. [6] was applied. ComBat applies an empirical Bayes approach, by pooling information across genes and shrinking the batch effect parameter toward the overall mean of the batch estimates across genes. This method has been claimed to be especially robust when handling multiple batches and batches of small samples sizes (<25) and has recently been shown to outperform other commonly used batch-adjustment methods [22].

After intraplatform ComBat batch adjustment, the samples from different batches were now intermingled, indicating that the ComBat method was successful in eliminating the batch effects (Figure 2(b)). To test if the preserved variation was related to true biological differences across tumor samples, molecular subtypes were classified according to PAM50 intrinsic molecular breast cancer subtypes. Numerous studies have shown that the main signals in gene expression datasets of breast cancers are associated with the five clinically relevant subtypes, termed intrinsic molecular breast cancer subtypes: basal-like, HER2-enriched, luminal A (lumA), and luminal B (lumB) [8, 18, 23–26]. In the 29K dataset prior to batch adjustments, no subgroupings related to these molecular subtypes were apparent. After ComBat adjustment, the molecular breast cancer subtypes were now clearly the main contributor to the overall variation (Figures 2(c) and 2(d)). This emphasizes the importance of distributing samples from different sample/treatment groups across batches; otherwise, adjustment for batch-to-batch variation would not have been possible.

**Figure 3:**
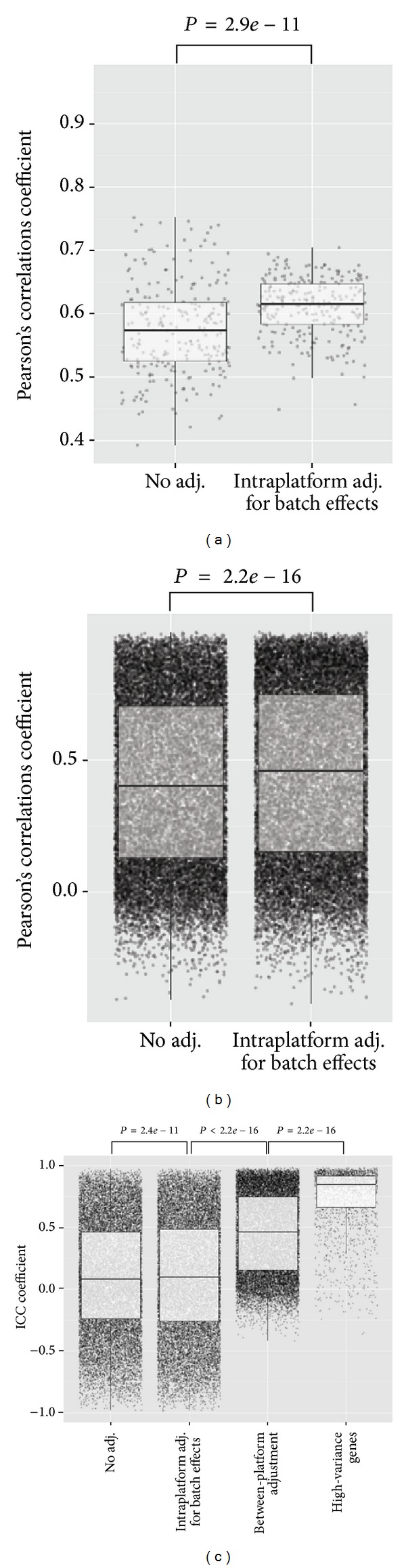
Correlation and ICC analysis. Box plots illustrating the pair-wise sample-sample correlation coefficients when no adjustment was applied and after intraplatform adjustment for batch effects using ComBat for all 243 sample pairs using the 18424 shared genes (a) and pair-wise gene-gene correlation coefficients for all 18424 gene-pairs across all 243 samples (b). Box plots illustrating effects of intraplatform, between-platform adjustment, and high-variance genes on the pair-wise gene-gene ICC coefficient measured for all 18424 gene-pairs across all 243 samples (c).

For a more quantitative evaluation on the effect of the adjustment of the 29K dataset, correlations between sample pairs and gene pairs from the two platforms were assessed by Person's correlation coefficient prior to and after the batch adjustment (Figures 3(a) and 3(b)). Person's correlation provides a measure of the strength and the linear relationship between two variables, even though they are not measured on the same quantitative scale. The pair-wise sample correlations between the unadjusted 29K dataset and the Agilent dataset ranged from 0.4 to 0.75 (median = 0.58). Adjusting the 29K dataset for batch effects introduced by the manufacturing procedure using ComBat as described above significantly improved the sample correlations (median = 0.62, *P* = 2.9*e* − 11), indicating that the overall gene expression patterns had become more alike. Similarly, correlations between gene pairs across all samples were calculated (Figure 3(b)). Correlations between the Agilent dataset and the unadjusted 29K dataset ranged from −0.4 up to 0.98 (median = 0.40). A significant improvement was gained when adjusting for intraplatform batch-to-batch differences introduced by the manufacturing procedure (median = 0.46, *P* < 2.2*e* − 16).

### 3.3. Pooling Microarray Datasets from Different Platforms

Pooling and integration of multiple microarray dataset are attractive to increase the number of sample in order to enhance statistical power. Because of the heterogeneous nature of microarray datasets, it is important to address the issue of cross-platform variation when combining dataset from multiple sources. In the current study, we were in the exceptional situation that a large number of samples had been analyzed on two different microarray platforms. This gave us a unique possibility to evaluate the significance of adjusting for interplatform differences by assessing the cross-platform agreement of the gene expression measurements. We chose to use the ComBat method for standardization of the two datasets. Intraclass correlation coefficients, ICC(1,1), were used as a measure of cross-platform agreement of the gene expression levels measured on the two platforms prior to and after interplatform adjustments. ICC is scale sensitive and provides an assessment of the consistency and reproducibility of quantitative measurements.

Interplatform adjustment led to a highly significant increase in ICC coefficients (median (prior to interplatform adj., only intraplatform adj.) = 0.1; median (after interplatform adj.) = 0.46; *P* < 2.2*e* − 16). To test if filtering of genes with low variance would improve correlation even further, we created a data subset retaining only genes with a standard deviation >0.3 across samples (2125 genes). When using only the high-variance genes, we gained additional improvement ICC coefficient (median = 0.84, *P* < 2.2*e* − 16), demonstrating that high-variance genes are more reproducibly measured across different microarray platforms. An ICC coefficient matrix between all possible sample pairs was generated. True for all sample pairs, the highest ICC coefficients were always observed when comparing the measurements of the same sample measured on the two platforms (data not shown).

A number of studies have been carried out to evaluate the correlation between data produced by different microarray platforms. Reports of both low and high reproducibility between different platforms exist [2, 27–29]. In a study by Kuo et al., they analyzed two RNA samples and examined gene pair correlations among five different platforms, both commercial and spotted. They found that correlations between platforms were in general good for most platforms with correlation coefficients ranging from 0.63 to 0.92 and the highest within platforms of the same type (single color platforms and dual color platforms). In addition, they showed that when probe sequences mapped to the same exon for a given gene, the measurements were found to be more similar across platforms [30].

Our results demonstrated that both intraplatform adjustment for batch effects and between-platform adjustments improve the reproducibility of the gene expression measurements. In addition, the analysis showed that by filtering out low-variance genes, we were able to enrich for genes that were highly reproducibly expressed across platforms, making high-variance genes particularly well suited as biomarkers or for building gene signatures.

### 3.4. Detection of Differentially Expressed Genes

**Figure 4:**
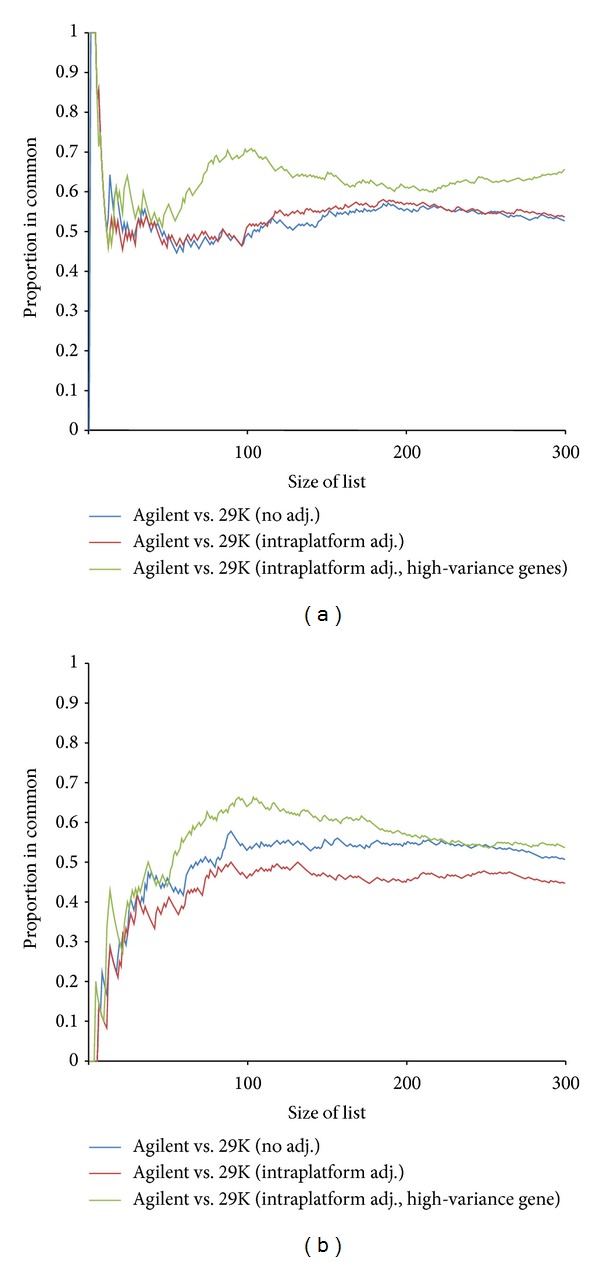
Detection of differentially expressed genes between ER-positive and ER-negative breast cancer samples (a) and between luminal A and luminal B samples (b), respectively. Significance analysis of microarrays (SAM) algorithm was applied to the Agilent dataset and to the 29K dataset both with and without ComBat intraplatform batch-effect adjustment, respectively. The resulting gene lists were then ranked according to their statistics. The plot shows the proportion of shared top genes between Agilent versus 29K unadj. (blue), Agilent versus 29K intraplatform adj. (red), and Agilent versus 29K intraplatform adj. using only high-variance genes (green), respectively.

A common application of gene expression profiling studies is to detect candidate genes that are differentially expressed between two or more groups. With the two datasets available derived from the same set of cancer samples, we were in a position to assess the agreement of differentially expressed genes across different platforms. For the purpose we applied the significance analysis of microarrays (SAM) algorithm, frequently used to identify differentially expressed genes between two groups. The samples were divided into estrogen receptor (ER) positive and negative samples. ER status was selected as it is a clinical used biomarker that is able to divide breast tumors into two clinically important and clearly distinguishable groups of cancers. The biological differences between ER+ and ER− samples are very strong and known to have a profound influence on the gene expression pattern. The SAM test was applied to the Agilent dataset and to the 29K dataset both with and without ComBat intraplatform batch-effect adjustment, respectively. The gene lists were then ranked according to their statistics. The proportion of shared top genes comparing Agilent versus 29K unadjusted and Agilent versus 29K intraplatform adjusted, respectively, is visualized in Figure 4(a) to provide a qualitative illustration of the size of overlap. Only minor effects of the ComBat adjustment were seen in the number of shared genes discriminating ER+ and ER− samples. To investigate whether more delicate differences in gene expression could be more sensitive to batch effects, we conducted the SAM analysis in order to identify genes discriminating between luminal A and luminal B subtypes (Figure 4(b)). Clearly, stronger improvements could be seen here. Larger overlaps in top genes discriminating between luminal A and luminal B samples were observed after the ComBat batch adjustment.

Furthermore, we sought to evaluate whether the high-variance genes also were among the most consistently expressed genes across platforms. By only including high-variance genes, the proportion of shared genes were clearly higher both in the ER+/ER− comparison and in the luminal A/B comparison, respectively (Figures 4(a) and 4(b)). Our results indicate that by filtering out low-variance genes prior to differential expression analyses, it was possible to enrich for genes that were consistently differentially expressed across platforms. Selecting valid and robust differential expressed genes is essential when identifying candidate genes for possible use as clinical biomarkers and gene signatures.

### 3.5. Prediction of Estrogen Receptor Status

**Figure 5:**
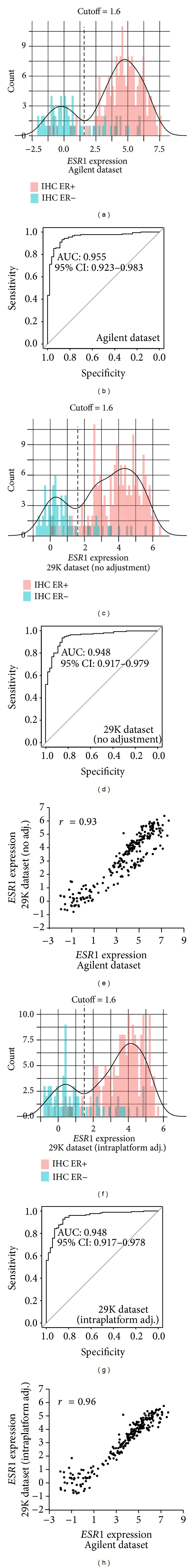
Density plot of* ESR1* gene expression levels and receiver operating characteristic (ROC) curves generated from samples with available immunohistochemical data, measured on the Agilent platform ((a) and (b)), 29K (no adjustments) ((c) and (d)), 29K (intraplatform adj.) and the 29K platform ((f) and (g)). Scatter plot visualizing the correlation between* ESR1* expression levels measured on the Agilent platform compared to 29K unadjusted and intraplatform adjusted measurements, respectively ((e), (h)).

For a detailed comparison of the gene expression pattern of a single gene, we choose the estrogen receptor (*ESR1*) due to its known clinical importance and the availability of immunohistochemical (IHC) ER expression data for the vast majority of the samples (231 out of 243). Density plots of the gene expression levels of the* ESR1* probe showed very clear bimodal distributions when measured on the Agilent platform, representing tumors with low and high expression of* ESR1*, respectively. The* ESR1*-low peaks show large overlap with the IHC ER negative tumors, whereas the* ESR1*-high peaks cover the majority of the IHC ER positive tumors. In the unadjusted 29K dataset, the* ESR1* measurement also displayed a bimodal distribution associated with the IHC ER status, although the* ESR1*-high peak was less distinct. ComBat intraplatform batch adjustment of the 29K corrected this, resulting in a bimodal distribution curve very similar to the Agilent measurements. The* ESR1* expression measurements on the Agilent and the 29K platform were highly correlated (Figure 5). Between the Agilent and 29K unadjusted datasets, the correlation coefficient obtained from* ESR1* was *r* = 0.93. ComBat batch adjustment further improved the correlation coefficient (*r* = 0.96). To evaluate the predictive power of* ESR1 *expression in relation to IHC ER status, we calculated the area under curve (AUC) of ROC curve analyses leading to very comparable results in the two datasets (AUC_agilent_ = 0.955, AUC_29K_ = 0.948). Using* ESR1* cutoff values obtained from the bimodal distribution (1.6 for all datasets), we obtained mean balanced accuracies of 89% and 88% for the Agilent dataset and the 29K datasets, respectively (Table 2). No differences between the batch adjusted and unadjusted datasets were observed. Fifty-four tumors were predicted as ER− and 185 tumors as ER+ in both datasets. Prediction results were discordant for only four tumors, indicating a strong association between the two prediction results (*P* < 2.2*e* − 16, Fisher's exact, Cramer's *V* association coefficient = 0.954) (Table 3).

The high levels of agreement between IHC-based ER status and ER status prediction and the high cross-platform concordance rate indicate that predictions of estrogen receptor status by microarray gene expression analysis are very robust. Because of its high discriminatory power, in a study by Li et al., the authors suggested that gene expression analysis might even be a better choice for assessing ER status compared to IHC-based analysis as it might be more sensitive compared to traditional IHC analysis [31]. They showed that up to 21% of patients of IHC-based negative ER status would have been classified as ER positive breast cancers by gene expression. Furthermore, in tamoxifen-treated cohorts, the gene expression measurements improved prediction of clinical outcome. Usually IHC-based ER-negative tumors are defined by staining in less than 10% of the cells. Gene expression profiles indicate that this cutoff should be adjusted in order to avoid false negative ER classification. Tumor heterogeneity might also contribute to the discordance between IHC and gene expression measurements.

### 3.6. Molecular Subtype Classification

**Figure 6:**
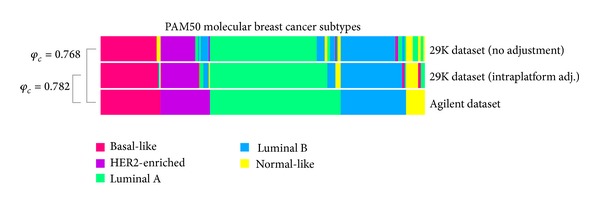
Comparison of the molecular subtype classification methods PAM50. Between Agilent and 29K (unadjusted) datasets, 203 samples were found to be concordant and 40 discordant (Cramer's *V* association coefficient = 0.768). Between Agilent versus 29K (intraplatform adjusted) the classifications resulted in 208 concordant and 35 discordant subtype predictions (Cramer's *V* association coefficient = 0.782).

For comparisons of molecular subtype classifications, we applied a commonly used classifier designed for single sample predictions, the 50-gene subtype classifier (PAM50) developed by Parker et al. [18]. PAM50 classifications were performed on the Agilent dataset, the unadjusted 29K dataset, and the intraplatform ComBat adjusted 29K dataset (Figure 6, Tables 4-5). As predictors based on centroids and correlation can be very sensitive, heatmaps using the PAM50 genes were generated in order to visually confirm that the subtype classification was reasonably correct (Supplementary Material, Figure S1-2, available online at http://dx.doi.org/10.1155/2014/651751). A strong association between subtype classifications was achieved when sample pairs across platforms were compared. Agilent versus 29K (unadjusted) classifications resulted in 203 concordant and 40 discordant subtype predictions (Cramer's *V* association coefficient = 0.768), and Agilent versus 29K (intraplatform adjusted) classifications resulted in 208 concordant and 35 discordant subtype predictions (Cramer's *V* association coefficient = 0.782). A high concordance rate was observed among tumors predicted as basal-like, whereas distinguishing luminal B from HER2-enriched and luminal A involves more disagreements. In a study by Waddell et al., they compared gene expression data from frozen breast tumor biopsies and FFPE tumor blocks. In 12 out of 15 cases, the FFPE/frozen sample pairs were concordant in relation to the molecular subtypes classified using the PAM50 classifier [32]. Our results indicate that PAM50 classification is a very robust method for prediction of molecular subtypes. The PAM50 genes were originally selected among genes that showed high variance across tumor. As shown above, high-variance genes tend to be more stable expressed across platforms, which may explain the robustness of our PAM50 subtype classification results across platforms supporting its validity as a clinical assay.

## 4. Conclusion

Using data from 243 breast cancer samples analyzed on two different microarray platforms, our* in-house* 29K array platform and Agilent SurePrint G3 microarray platform, we demonstrated the importance of detecting and tackling batch-related biases within datasets prior to data analysis. We have demonstrated how tools such as ComBat are suitable to successfully overcome systematic technical variations in order to unmask essential biological signals. Batch adjustment was found to be particularly valuable in the detection of more delicate differences in gene expression. Furthermore, the study shows that prober adjustment is essential for optimal integration of gene expression data obtained from multiple sources. The data also demonstrate that high-variance genes are highly reproducibly expressed across platforms, making them particularly well suited as biomarkers or for building gene signatures. Predictions of estrogen-receptor status and molecular breast cancer subtypes were found to be highly concordant. In conclusion, the study emphasizes the importance of utilizing proper batch adjustment methods to reduce systematically technical bias when analyzing and integrating data from different batches and microarray platforms and by selecting high-variance genes it is possible to enrich for highly reproducible genes.

## Supplementary Material

Supplementary Figure S1: shows the PAM50 classification and associated heatmap visualizing the PAM50 genes using the Agilent dataset.
Supplementary Figure S2: shows the PAM50 classification and associated heatmap visualizing the PAM50 genes using the 29K (unadjusted) dataset.

## Figures and Tables

**Table 1:** Overview of fabrication batches of 29K arrays.

Batch	Number of arrays
Batch 03	46
Batch 05	8
Batch 06	67
Batch 07	12
Batch 08	50
Batch 09	60

	243

**Table 2:** Prediction of ER status by *ESR1* expression levels in the Agilent dataset and the 29K dataset.

Platform	Number of samples	Cutoff	Sensitivity (TP)	Specificity (TN)	Accuracy
Agilent dataset	177 vs. 54	1.6	0.82 (44)	0.96 (169)	0.89
29K dataset (no adjustment)	177 vs. 54	1.6	0.82 (44)	0.94 (167)	0.88
29K dataset (intraplatform adj.)	177 vs. 54	1.6	0.82 (44)	0.94 (167)	0.88

**Table 3:** Association between ER prediction results by the two platforms.

Agilent/29K	ER−	ER+
ER−	54	1
ER+	3	185

**Table 4:** Association between PAM50 molecular subtype classifications in the Agilent and the 29K dataset (unadjusted).

Agilent/29K	Basal-like	HER2-enriched	Luminal A	Luminal B	Normal-like
Basal-like	**42**	0	1	1	0
HER2-enriched	0	**27**	0	1	0
Luminal A	0	3	**83**	4	6
Luminal B	0	7	10	**43**	0
Normal-like	3	0	4	0	**8**

**Table 5:** Association between PAM50 molecular subtype classifications in the Agilent and the 29K dataset (intraplatform adjusted).

Agilent/29K	Basal-like	HER2-enriched	Luminal A	Luminal B	Normal-like
Basal-like	**42**	0	0	1	1
HER2-enriched	1	**25**	0	1	1
Luminal A	0	2	**84**	5	5
Luminal B	1	2	8	**49**	0
Normal-like	3	0	3	1	**8**
