# Supplementary material for: Microarray-Based RNA Profiling of Breast Cancer: Batch Effect Removal Improves Cross-Platform Consistency
Source: Biomed Res Int. 2014 Jul 3;2014:651751. doi: 10.1155/2014/651751 (PMC4101981; doi:10.1155/2014/651751)

Figure S1. Heatmap visualizing the PAM50 genes on the Agilent dataset. Color coding represents PAM50 subtype classification results: Red: Basal; Yellow: HER2-enriched; Green: Luminal-A; Blue: Luminal-B; Purple: Normal-like.

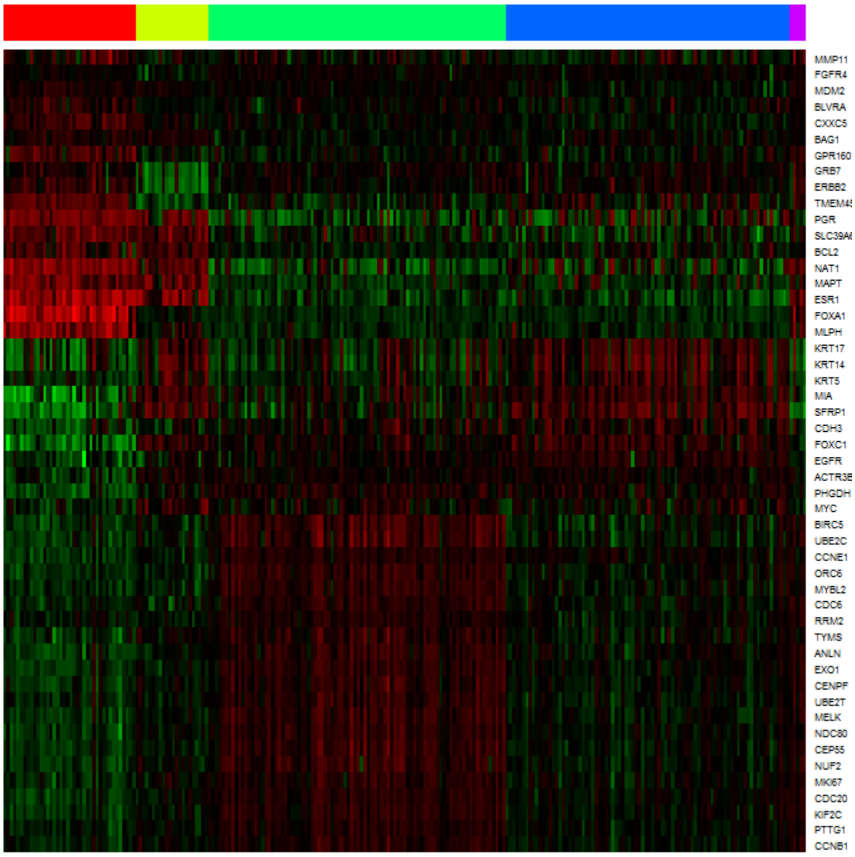

Figure S2. Heatmap visualizing the PAM50 genes on the 29K unadjusted dataset. Color coding represents PAM50 subtype classification results: Red: Basal; Yellow: HER2-enriched; Green: Luminal-A; Blue: Luminal-B; Purple: Normal-like.

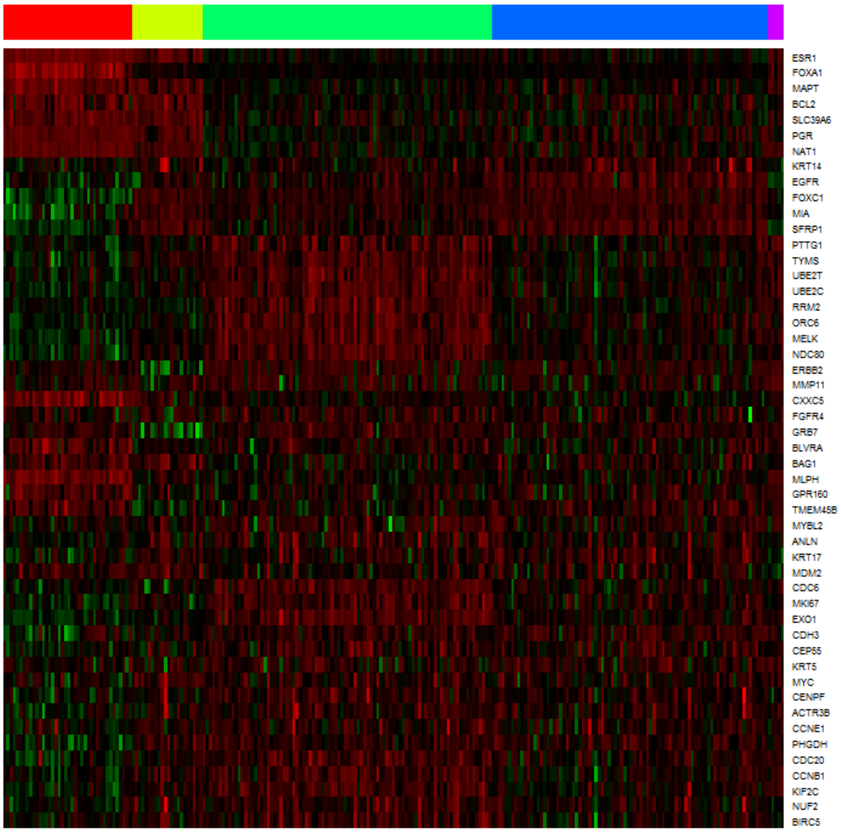

Supplement: Supplementary file 1 — Supplementary Figure S1: shows the PAM50 classification and associated heatmap visualizing the PAM50 genes using the Agilent dataset. Supplementary Figure S2: shows the PAM50 classification and associated heatmap visualizing the PAM50 genes using the 29K (unadjusted) dataset. [file 651751.f1.pdf]
